# Cancer and the junkyard chromosome: how repeat DNA sequence on chromosome 19 influences risk of malignant disease

**DOI:** 10.18632/oncotarget.25873

**Published:** 2018-08-10

**Authors:** Anna M. Rose

**Affiliations:** MRC Weatherall Institute of Molecular Medicine, John Radcliffe Hospital, Oxford, UK

**Keywords:** MSR1, repeat element, breast cancer, prostate cancer, gene regulation

Although all of the chromosomes are unique, it could be said that chromosome 19 is the *most* unique. Of all the chromosomes, it carries the highest density of genes - more than double the average gene content - and it is also unusually rich in clustered gene families, CpG islands and repetitive DNA elements [1]. The repeat content of chromosome 19 is approximately 55% - this being 10% higher than the genome-wide average - and comprised of mainly Alu and LINE elements [1]. Amongst these is one unusual element: MSR1, a 36-38bp minisatellite sequence that is predominantly located at chromosome 19q13, but occurs in degenerate form across the genome [2, 3].

Minisatellites and microsatellites are variable number tandem repeats, and regions containing these repeats are highly unstable. Microsatellites (short repetitive tandem sequences) have been known to affect gene expression through change of sequence length within promoters and other *cis*-regulatory regions, and this has important implications for human malignancy [4]. Recently, a similar role for MSR1 minisatellite sequences has been described, with important consequences for risk of non-familial breast cancer and prostate cancer.

In their recent work, Rose *et al.* demonstrate that MSR1 repeats (i) are enriched in regulatory regions, (ii) alter gene expression through copy number variation (CNV), and (iii) influence risk of cancer [3]. In particular, work was focussed on the kallikrein locus - a cluster of serine-protease genes with well-described role in endocrine malignancies, such as breast, prostate and ovarian carcinoma.

It was demonstrated that the kallikrein locus had a large number of MSR1 repeat clusters and that these were frequently located in regulatory regions of the genes, such as the promoter or 5’/3’ untranslated regions (UTR). One cluster was identified within the 3’ UTR of *KLK14*, and it was found to be highly polymorphic in UK and Australian populations - with 6-13 copies being normal variants. The majority of individuals, however, had either 11 copies (79.8-85.3% alleles) or 9 copies (14.1-17.1% alleles). Crucially, it was shown that both elements could act as an enhancer for a basic promoter, but the activity of the 9-copy allele was much stronger than the 11-copy allele. It was hypothesised, therefore, that the 9-copy alleles might drive higher expression of *KLK14 in vivo* and this might influence risk of endocrine cancers, given the frequent over-expression of kallikreins in these tumours. In a case-control cohort, the group found that the 9-copy MSR1 allele conferred an increased risk of 1.21-3.51 times for all non-familial breast cancer, but - strikingly - 1.7 to 5.3 times increased risk in early-onset disease. The 9-copy allele was also found to be associated with increased risk of prostate cancer in an independent population.

It appears, then, that regulation of gene expression by MSR1 plays an important role at *KLK14* - and that this has clinically relevant implications. The MSR1 polymorphism at *KLK14* is the highest influencing risk factor identified to-date in non-familial breast cancer, and the risk ratio for prostate cancer was also clinically useful. However, the potential scope of the work is much larger. The group found that there are a large number of MSR1 clusters within the kallikrein locus and a number of these were shown to demonstrate CNV. It is predicted that a combinatorial model of MSR1 genotypes across the kallikrein locus might be used to produce a robust stratification of endocrine cancer risk. This would hopefully lead to prediction of those at highest risk of non-familial disease, and enrolment in effective screening and prevention programmes. Perhaps of even greater significance, there are hundreds of genes across chromosome 19 that are potentially controlled by MSR1s and many of these have been associated with cancer risk or prognosis in genome-wide association studies (Table 1). Detailed assessment of CNV at these loci (and the effect of CNV on disease risk) could lead to a precise and highly clinically-relevant model of genetic risk for various common cancers that would lead directly to patient benefit, with secondary beneficial outcomes for the healthcare economy.

**Table 1:** GWAS association with risk of malignancy for genes putatively regulated by MSR1 from two GWAS databases, and cancers associated with dysregulation of kallikrein genes

Database	Gene	Associated cancers
NHGRI-EBI GWAS Catalog	*BCL3*	Oesophageal adenocarcinoma
	*BRSK1*	Breast cancer
	*CA11*	Elevated serum carcinoembryonic antigen levels in patients with colorectal cancer
	*CYP2A6*	Lung sqaumous cell carcinoma, lung adenocarcinoma
	*DBP*	Elevated serum carcinoembryonic antigen levels in patients with colorectal cancer
	*FUT1*	Elevated serum carcinoembryonic antigen levels in patients with colorectal cancer
	*FUT2*	Lung adenocarcinoma
	*GMFG*	Lung adenocarcinoma
	*KCNN4*	Breast cancer
	*KLK2*	Prostate cancer
	*KLK3*	Prostate cancer
	*LRFN1*	Lung adenocarcinoma
	*LYPD5*	Breast cancer
	*SBK2*	Lung adenocarcinoma
	*SSC5D*	Lung adenocarcinoma
	*SULT2B1*	Elevated serum carcinoembryonic antigen levels in patients with colorectal cancer
	*TARM1*	Small cell lung cancer
	*ZNF283*	Breast cancer
GWAS central (-log ≥2)	*ACPT*	Hodgkin Lymphoma
	*ACTN4*	Breast cancer
	*CPT1C*	Prostate cancer
	*CYP2S1*	Breast cancer
	*HSD17B14*	Prostate cancer
	*KLK4*	Breast cancer
	*RUVBL2*	Breast cancer
	*TP73*	Breast cancer
	*TULP2*	Breast cancer
Kontas and Scorilas [8]	*KLK1*	RCC
	*KLK2*	Prostate and ovarian cancers
	*KLK3 (PSA)*	Prostate, ovarian, and breast cancers
	*KLK4*	Prostate, ovarian, and breast cancers
	*KLK5*	Prostate, testicular, ovarian, colorectal, breast, and lung cancers; HNSCC
	*KLK6*	RCC; ovarian, uterine, colorectal, gastric, and lung cancers
	*KLK7*	RCC; ovarian, cervical, colorectal, breast, and lung cancers; HNSCC
	*KLK8*	Ovarian, uterine, cervical, and lung cancers; HNSCC
	*KLK9*	Ovarian and breast cancers
	*KLK10*	Testicular, ovarian, uterine, colorectal, gastric, breast, and lung cancers; RCC; HNSCC; ALL
	*KLK11*	Prostate, testicular, and ovarian cancers; RCC; HNSCC
	*KLK12*	Breast cancer
	*KLK13*	Testicular, ovarian, colorectal, gastric, breast, and lung cancers
	*KLK14*	Prostate, testicular, ovarian, colorectal, breast, and lung cancers
	*KLK15*	Prostate, ovarian, and breast cancers

Abbreviations: HNSCC - head and neck squamous cell carcinoma; RCC - renal cell carcinoma; ALL - acute lymphoblastic leukaemia.

Many questions remain unanswered regarding MSR1 and further research is required. First and foremost, it is unclear how CNV of MSR1 alters gene expression. It is possible that the elements affect transcription factor binding or influence interaction of the transcriptional machinery with the gene promoter. It is also plausible that MSR1 repeats are a target for epigenetic regulation, such as methylation. MSR1 repeats might also form non-canonical or secondary DNA structures, thereby affecting expression of local genes. Functional work will be critical in understanding the mechanism of action - and whether this can be targeted by novel anti-cancer therapies. Secondly, it seems plausible that hypermutation of MSR1 copy number might occur within tumours, further promoting gene dysregulation. Hypermutation of other repetitive elements - such as microsatellites - is well described in many cancer types (particularly colorectal, endometrial, and gastric adenocarcinomas) and is both predictive and prognostic of disease [5]. A further role for MSR1 in aberrant gene expression in cancer was suggested by work which showed that MSR1 sequence is included within a KLK4 sense-antisense chimera in prostate cancer cells, perhaps influences expression of the abnormal transcript [6].

It is also important to ask whether the degenerate MSR1 sequences found on chromosomes other than chromosome 19 are functional. Again, study of the kallikreins might shed light on this fascinating question. In humans, kallikrein genes fall into two major categories: plasma and tissue; there is only one plasma kallikrein - *KLKB1* - which is encoded at chromosome 4q35 [7]. Intriguingly, there are only 4 occurrences of MSR1 on chromosome 4 - but one of the clusters is associated with *KLKB1*. This implies that there has been selection pressure to maintain the MSR1 element after insertion of the kallikrein gene onto chromosome 4 and so, perhaps, suggests retained molecular function of the degenerate element.

It is clear that MSR1 repeats are a widespread regulator of gene expression and that this prototypical “junk DNA” element potentially influences many cancer types. Assessment of various MSR1 clusters will allow development of tools for screening, diagnosis and prognostication for malignancy, with the aim of prevention or early diagnosis. Perhaps, MSR1 will be a new therapeutic target, allowing development of a new class of chemotherapeutic agents. This is just the start of the MSR1 story and we will watch with great interest to see how it unfolds.
